# North Korean defectors with PTSD and complex PTSD show alterations in default mode network resting-state functional connectivity

**DOI:** 10.1192/bjo.2023.636

**Published:** 2024-01-05

**Authors:** Byung-Hoon Kim, Jiwon Baek, Ocksim Kim, Hokon Kim, Minjeong Ko, Sang Hui Chu, Young-Chul Jung

**Affiliations:** Department of Psychiatry, Yonsei University College of Medicine, Seoul, Republic of Korea; and Institute of Behavioral Sciences in Medicine, Yonsei University College of Medicine, Seoul, Republic of Korea; Mo-Im Kim Nursing Research Institute, Yonsei University College of Nursing, Seoul, South Korea; Department of Nursing, Yonsei University College of Nursing and Brain Korea 21 FOUR Project, Seoul, South Korea; Department of Nursing, Yonsei University College of Nursing, Seoul, South Korea; and Brain Korea 21 FOUR Project, Seoul, South Korea; Department of Nursing, Yonsei University College of Nursing, Seoul, South Korea; Mo-Im Kim Nursing Research Institute, Yonsei University College of Nursing, Seoul, South Korea; and Department of Nursing, Yonsei University College of Nursing, Seoul, South Korea

**Keywords:** North Korean defectors, PTSD, complex PTSD, functional connectivity, default mode network

## Abstract

**Background:**

North Korean defectors (NKDs) have often been exposed to traumatic events. However, there have been few studies of neural alterations in NKDs with post-traumatic stress disorder (PTSD) and complex PTSD (cPTSD).

**Aims:**

To investigate neural alterations in NKDs with PTSD and cPTSD, with a specific focus on alterations in resting-state functional connectivity networks, including the default mode network (DMN).

**Method:**

Resting-state functional connectivity was assessed using brain functional magnetic resonance imaging in three groups of NKDs: without PTSD, with PTSD and with cPTSD. Statistical tests were performed, including region of interest (ROI)-to-ROI and ROI-to-voxel analysis, followed by *post hoc* correlation analysis.

**Results:**

In the ROI-to-ROI analysis, differences in functional connectivity were found among the components of the DMN, as well as in the thalamus and the basal ganglia. Right hippocampus–left pallidum and right amygdala–left lingual gyrus connectivity differed between groups in the ROI-to-voxel analysis, as did connectivity involving the basal ganglia. The *post hoc* analysis revealed negative correlations between Coping and Adaptation Processing Scale (CAPS) score and left posterior cingulate cortex–right pallidum connectivity and between CAPS score and right putamen–left angular gyrus connectivity in the control group, which were not observed in other groups.

**Conclusions:**

The results suggest that there are alterations in the functional connectivity of the DMN and the limbic system in NKDs with PTSD and cPTSD, and that these alterations involve the basal ganglia. The lower correlations of CAPS score with right basal ganglia–DMN functional connectivity in patients compared with controls further implies that these connectivities are potential targets for treatment of PTSD and cPTSD.

Refugees are displaced from their homelands owing to external factors related to survival. Forced displacement as a result of armed conflict, political persecution or natural disasters causes significant psychological stress. Refugees commonly experience adversities before and during their migration, and living conditions after migration can be harsh and stressful. The high prevalence of post-traumatic stress disorder (PTSD) among refugees is thought to be related to their exposure to traumatic events before, during and after their displacement across national and cultural borders.^1^ As the total number of refugees has grown rapidly over the past few decades, reaching over 27.1 million individuals worldwide by the end of 2021,^2^ the mental health needs of refugees, who are vulnerable to developing psychiatric symptoms and mental illnesses such as PTSD,^3^ have been addressed to ensure timely diagnosis, treatment and rehabilitation.

So far, approximately 30 000 North Korean defectors (NKDs), a subgroup of refugees, have fled North Korea and entered South Korea. South and North Korea have been separated for about 80 years. Although they still have the same ethnicity and language, differences in culture, ideology, government regulations and socioeconomic status have grown significantly, making it difficult for NKDs to adapt and settle in South Korea.^4^ A recent large-scale analysis that assessed over 22 000 NKDs with propensity score matching against 90 000 people in South Korea indicated that NKDs have higher risk of developing mental disorders than South Koreans, with a hazard ratio of 2.12.^5^ Unsurprisingly, PTSD had the highest hazard ratio of 3.10, suggesting harmful effects of the traumatic experiences of displaced individuals, as emphasised in previous studies.^6^^,^^7^ Difficulties in adapting to a new environment can be worsened by symptoms of PTSD in refugees.^8^^,^^9^ Currently, reducing mental health problems in NKDs is a socially important issue in South Korea.

Here, it should be noted that the classical definition of trauma for the diagnosis of PTSD is a discretised exposure to actual or threatened death or serious harm.^10^ However, NKDs often experience prolonged exposure to non-physical trauma such as political–ideological and family-related trauma.^6^ The different role of prolonged and repeated trauma in the development of PTSD symptoms has been acknowledged in psychiatric diagnoses. To address this issue, the ICD-11 was officially updated to define complex PTSD (cPTSD) as a disorder caused by multiple and sustained traumas, distinct from PTSD.^11^ Our previous study showed that quality of life (QoL) among NKDs with cPTSD symptoms was lower than that in NKDs with low levels of symptoms or even with PTSD.^12^ However, to date, only a few studies have focused on cPTSD in refugees or, more specifically, in NKDs.^13^

With the development of neuroimaging methods, it has been shown that psychological trauma affects the neurobiological system of the human brain.^14^^,^^15^ The brain regions that are most often associated with PTSD include the amygdala, hippocampus and medial prefrontal cortex.^16^ These three regions are hypothesised to form a neurocircuitry that is centred on the amygdala, the limbic system responsible for fear responses.^17^ Specifically, the amygdala takes inputs to process and learn about fear and sends output signals to other brain regions, such as the hypothalamus, thalamus, and brainstem, to activate the fear response.^16^ When the amygdala responds to fear, the medial prefrontal cortex and the hippocampus work together to downregulate the level of reaction to an appropriate range.^18^^,^^19^ Neuroimaging studies have shown that in patients with PTSD, the amygdala is hyperactivated in response to fear stimuli, and the hippocampus shows reduced volume when compared with that of healthy controls.^20^^–^^24^

Another recent perspective extends the neurocircuitry hypothesis to functional network-level disruptions in patients with PTSD.^24^ The default mode network (DMN) is one of the most important functional connectivity networks activated at rest and involves the posterior cingulate cortex (PCC), precuneus, medial prefrontal cortex and angular gyrus.^25^ The DMN is known to have a role in self-referential thinking and memory recall,^26^ and disruption of the DMN has been found in patients with psychiatric disorders including major depressive disorder,^27^ schizophrenia^28^ and Alzheimer's disease.^29^ Evidence from resting-state functional magnetic resonance imaging (fMRI) studies suggests that patients with PTSD exhibit alterations of the DMN. For example, combat veterans with PTSD exhibit reduced functional connectivity between the PCC and the hippocampus.^30^ Although some structural neuroimaging studies have focused on the effects of PTSD on structural changes of the brain specific to NKDs,^31^^,^^32^ not much is known about the changes in the resting-state functional neural network involved in PTSD in NKDs. Furthermore, no previous research has specifically investigated any difference in neural findings between PTSD and cPTSD in traumatised individuals.

Although most people experience at least one traumatic event in their lives, not all develop clinically significant symptoms or disorders such as PTSD. The Roy adaptation model is one of the most widely used models conceptualising the coping process and delineating cognition and regulator subsystems.^33^ The Coping and Adaptation Processing Scale (CAPS) is a psychometric scale built upon the Roy adaptation model that can be used to evaluate style and quantify one's capacity for coping.^34^ Given that coping and adaptation processing is a crucial part of recovery after traumatic events and could be related to clinical outcomes,^35^^,^^36^ we used the CAPS to investigate the neural correlates of coping capacity in patients with PTSD and cPTSD.

In this study, we aimed to study the neural underpinnings of PTSD and cPTSD among NKDs. More specifically, we measured functional neural activity using fMRI during rest in a group of NKDs diagnosed with PTSD or cPTSD compared with an asymptomatic group and analysed alterations in resting-state functional connectivity. Here, we tested three hypotheses: (a) that functional connectivity related to the limbic system, especially the amygdala and hippocampus, would be altered in patients with PTSD or cPTSD; (b) that functional connectivity related to the DMN, especially the PCC, would be altered in patients with PTSD or cPTSD; and (b) coping capacity would show different correlation patterns with functional connectivity in both patients with PTSD and those with cPTSD compared with people without PTSD.

## Method

### Participant recruitment and evaluation

**Fig. 1:**
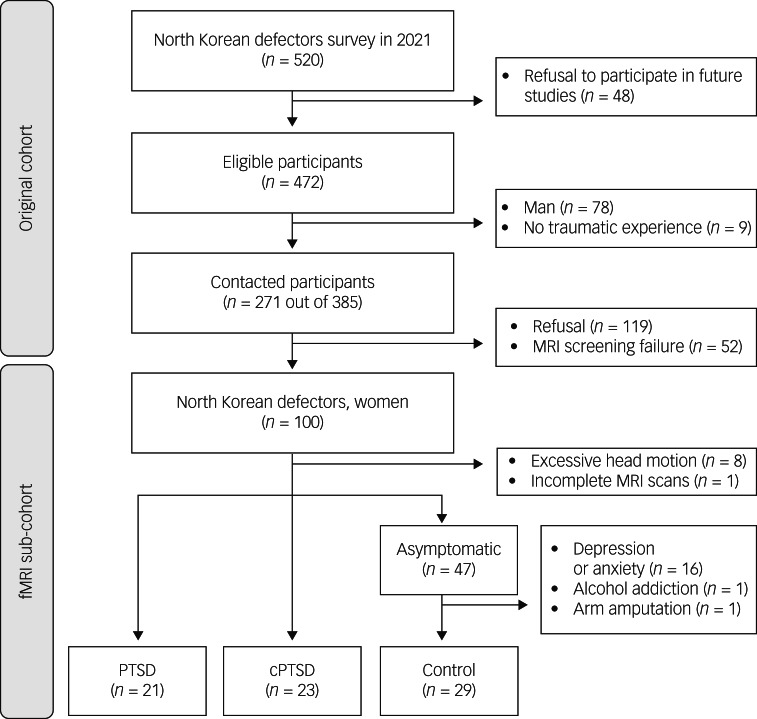
Flow diagram of participant recruitment and inclusion. MRI, magnetic resonance imaging; fMRI, functional magnetic resonance imaging; PTSD, post-traumatic stress disorder; cPTSD, complex post-traumatic stress disorder.

Participants were recruited from the original cohort of 520 NKDs who participated in an online survey in 2021.^12^ In the current study, we recruited only women, accounting for 70% of NKDs, to exclude biological variations in the brain by sex or related hormones. Initially, 271 of the 385 eligible cohort participants were contacted for the follow-up study after excluding men (*n* = 78) and those who reported no history of traumatic events (*n* = 9). Of the 271 patients, 119 refused to participate, and 52 had pre-existing conditions that precluded an MRI scan (e.g. metal implants, claustrophobia, possible pregnancy). Finally, 100 NKD women participated in the current study. Nine participants were excluded because of excessive head motion (*n* = 8) or incomplete MRI scans (*n* = 1), and 18 were additionally excluded from the asymptomatic group who had clinically diagnosed depressive or anxiety disorders (*n* = 16), alcohol addiction (*n* = 1) or arm amputation (*n* = 1). The final sample consisted of 73 NKD women residing in South Korea, all of whom had been diagnosed with PTSD (*n* = 21) or cPTSD (*n* = 23) or were asymptomatic controls (*n* = 29). A flowchart of the participant recruitment process is shown in Fig. 1.

For the final participants, a clinical diagnosis of PTSD or cPTSD was made through a psychiatric interview with a skilled psychiatrist (Y.C.J.). The participants also completed self-report psychometric scales, including the Korean version of the CAPS,^37^ which evaluates coping styles in response to stressful events. The CAPS is a four-point Likert scale with 15 items; higher scores reflect better coping skills. Overall QoL was measured by one item from WHOQOL-BREF asking for the participant's overall perception of QoL with a scoring range of 0–4.

The authors assert that all procedures contributing to this work comply with the ethical standards of the relevant national and institutional committees on human experimentation and with the Helsinki Declaration of 1975, as revised in 2008. All procedures involving human subjects/patients were approved by the ethical review boards of the Severance Hospital Institutional Review Board (no. 4-2021-1520). Formal written informed consent was obtained from all participants in this study.

### Neuroimage data acquisition and pre-processing

A Philips Ingenia CX 3.0T MRI scanner (Philips, Amsterdam, The Netherlands) was used to acquire the neuroimaging data. The participants were instructed to rest with their eyes open during the entire scanning process. T1-weighted structural images were obtained using the SENSE parallel imaging protocol with the following parameters: repetition time, 9.9 ms; echo time, 4.6 ms; flip angle, 8°; number of slices, 170; slice thickness, 1 mm; and matrix size, 512 × 512. An echo planar image sequence with the following parameters was used to acquire the functional images: repetition time, 2000 ms; echo time, 22 ms; flip angle, 90°; number of slices, 49; slice thickness, 3 mm; and matrix size, 80 × 80. The acquired images were pre-processed on a Linux workstation using fMRIPrep (v. 21.0.2),^38^ which is based on Nipype.^39^ Pre-processing of T1-weighted structural images included brain extraction, tissue segmentation, surface extraction and spatial normalisation, whereas pre-processing of resting-state functional images included head-motion correction, slice-timing correction and co-registration. Further details of the pre-processing of neuroimaging data are provided in the Supplementary Material available at https://doi.org/10.1192/bjo.2023.636.

### Functional connectivity analysis

The pre-processed structural and functional images for the 73 participants were imported into the Conn toolbox (v. 21.a) in MATLAB (v. 2022a) for analysis of resting-state functional connectivity.^40^ Two types of analysis were performed: region of interest (ROI)-to-ROI and ROI-to-voxel. For the ROI-to-ROI analysis, the time series of 116 ROIs defined from the Automated Anatomical Labeling (AAL) Atlas^41^ were first extracted by averaging the BOLD signal of voxels within each ROI. Functional connectivity was defined as the Pearson's correlation coefficient between every possible pair of the 116 ROI time series across time, subjected to Fisher's z-transformation. Using this procedure, a 116 × 116 resting-state functional connectivity matrix was obtained for each participant, on which the ROI-to-ROI statistical analysis was conducted. For the ROI-to-voxel analysis, we selected four ROIs from the AAL Atlas, including the amygdala and hippocampus, in both hemispheres. Functional connectivity between the selected ROI and every voxel within the resting-state fMRI data was computed, and this voxel-wise functional connectivity was used for the ROI-to-voxel statistical analysis.

### Statistical analysis

For demographic factors, differences between groups were evaluated using one-way analysis of variance (ANOVA). For the ROI-to-ROI analysis, differences in functional connectivity were analysed using ANOVA with cluster-level multivariate pattern analysis omnibus test corrected for multiple comparisons with false discovery rate (FDR) *P*-FDR < 0.05 and uncorrected connection-level threshold *P* < 0.05 following the standard approach from ref.^42^ and the Conn toolbox. For the ROI-to-voxel analysis, differences between the groups were also analysed with ANOVA with an uncorrected voxel-level threshold of *P* < 0.001 and cluster-level threshold of *k* ≥ 10. For the functional connectivities that showed significant results in the ANOVA, a *post hoc* Tukey test was performed to further identify the differences between each pair in the three groups. To test the relationship between functional connectivity and trauma-related characteristics, the *post hoc* Pearson's correlation between functional connectivity strength and CAPS was evaluated per group. All statistical analyses were conducted with Python 3.8.5 using the ‘pandas’ and ‘pingouin’ packages, except for the group difference test for functional connectivity, which was performed with the Conn toolbox.

## Results

### Demographics and psychometric scales

Demographic characteristics and psychometric scale scores for each group are summarised in Table 1. No between-group differences were found for age, years of residency in South Korea or education level. No significant differences were found in QoL scores among the control, PTSD and cPTSD groups; however, CAPS scores showed significant differences among groups (F = 6.658, *P* = 0.002).

**Table 1:** Demographic characteristics and psychometric scale scores of study participants; mean values for each group are reported, with the standard deviation denoted in parentheses

	Control (*n* = 29)	PTSD (*n* = 21)	cPTSD (*n* = 23)	F	*P*
Age, years	44.21 (10.26)	46.29 (9.28)	47.52 (11.62)	0.672	0.514
Years of residence in South Korea	11.86 (4.89)	13.10 (3.89)	11.65 (4.87)	0.630	0.536
Overall QoL	3.48 (0.95)	3.24 (0.70)	3.13 (0.81)	1.205	0.306
CAPS score	43.13 (3.91)	39.71 (5.37)	37.35 (7.73)	6.658	0.002

PTSD, post-traumatic stress disorder; cPTSD, complex post-traumatic stress disorder; QoL, quality of life; CAPS, Coping and Adaptation Processing Scale.

### Functional connectivity analysis

**Fig. 2:**
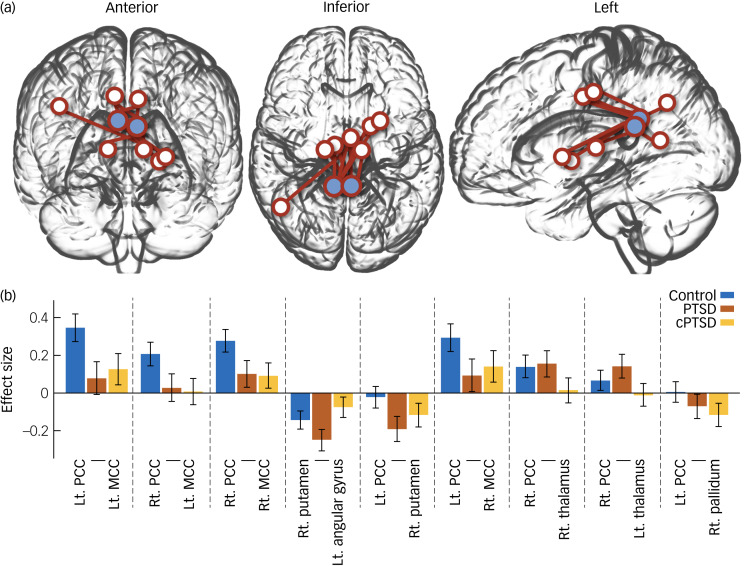
(a) Glass brain plot of region of interest (ROI)-to-ROI analysis results showing significant group differences. Blue circles indicate bilateral posterior cingulate cortices. (b) Bar plot of ROI-to-ROI analysis of variance results. PTSD, post-traumatic stress disorder; cPTSD, complex post-traumatic stress disorder; Lt, left; Rt, right; PCC, posterior cingulate cortex; MCC, middle cingulate cortex.

**Fig. 3:**
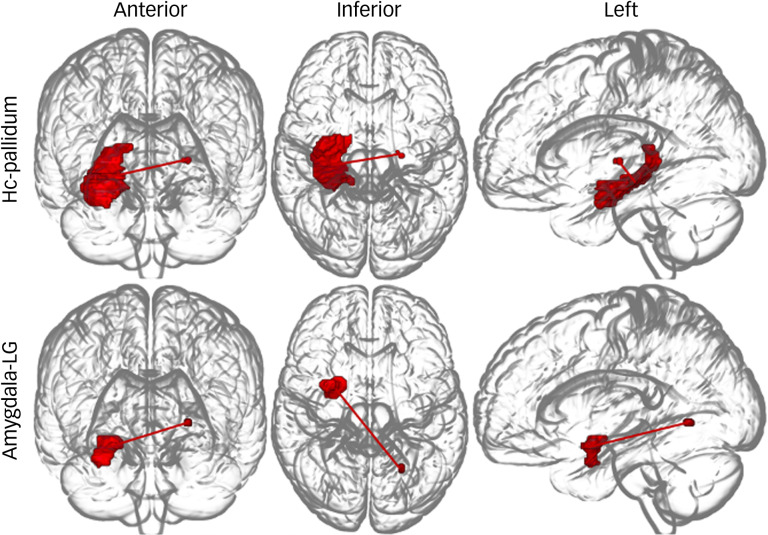
Glass brain plot of ROI-to-voxel analysis results showing significant group differences. ROI, region of interest; Hc, hippocampus; LG, lingual gyrus.

ROI-to-ROI functional connectivity analysis revealed nine pairs of functional connectivity across the bilateral PCC, bilateral middle cingulate cortex (MCC), bilateral thalamus, right putamen, right pallidum and left angular gyrus; the nine pairs of ROIs differed significantly among groups (Fig. 2). Among the results, all functional connectivity, except that between the right putamen and left angular gyrus, was related to the PCC (Table 2). Functional connectivity between the PCC and MCC showed the highest F-statistic among all connections. From the ROI-to-voxel analysis, significant group differences in functional connectivity were found between the right hippocampus and a region within the left pallidum (*P* < 0.001, *k* = 10) and between the right amygdala and the left lingual gyrus (*P* < 0.001, *k* = 12; Fig. 3). No significant results were found for functional connectivity with the left hippocampus and left amygdala as seed ROIs.

**Table 2:** Statistics from analysis of variance of functional connectivity between groups

Functional connectivity	F	*P*
Lt. PCC–Lt. MCC	9.45	<0.001
Rt. PCC–Lt. MCC	7.96	0.001
Rt. PCC–Rt. MCC	7.58	0.001
Rt. putamen–Lt. angular gyrus	7.23	0.001
Lt. PCC–Rt. putamen	5.29	0.007
Lt. PCC–Rt. MCC	4.91	0.010
Rt. PCC–Rt. thalamus	3.88	0.025
Rt. PCC–Lt. thalamus	4.33	0.017
Lt. PCC–Rt. pallidum	3.17	0.048

Lt, left; Rt, right; PCC, posterior cingulate cortex; MCC, middle cingulate cortex.

### *Post hoc* statistical analysis

**Fig. 4:**
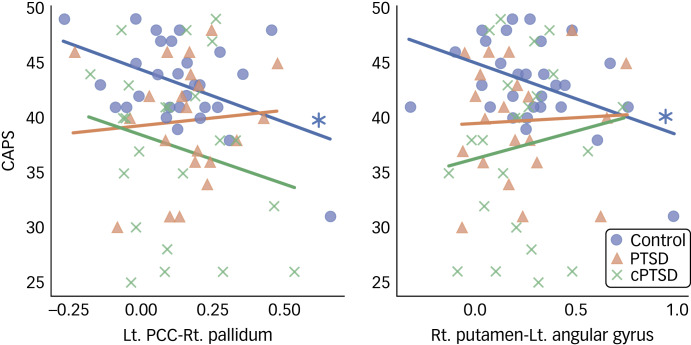
Scatter plot of significant *post hoc* correlation analysis results. CAPS, Coping and Adaptation Processing Scale; PTSD, post-traumatic stress disorder; cPTSD, complex post-traumatic stress disorder; Lt, left; Rt, right; PCC, posterior cingulate cortex.

The *post hoc* Tukey test for the ROI-to-ROI analysis results demonstrated significantly decreased functional connectivity in patients with cPTSD between the left PCC and right putamen compared with the control group (*t* = 2.641, *P* = 0.027) and between the right PCC and bilateral MCC compared with the PTSD group (left: *t* = 2.664, *P* = 0.026; right: *t* = 2.770, *P* = 0.019). No other significant group difference was found by *post hoc* Tukey test for the ROI-to-ROI analysis. Correlation analysis revealed significant negative correlations of CAPS scores with functional connectivity between the left PCC and right pallidum (*r* = −0.446, *P* = 0.015) and between the right putamen and left angular gyrus in the control group (*r* = −0.433, *P* = 0.0019; Fig. 4). No significant differences were found in the *post hoc* statistical analysis of the ROI-to-voxel results.

## Discussion

In this study, we investigated resting-state functional connectivity in traumatised NKDs. Disruption of the functional connectivity network grounded in the bilateral PCC was found in the ROI-to-ROI analysis, suggesting abnormal changes in the DMN in NKDs with cPTSD. ROI-to-voxel analysis showed altered functional connectivity between the right hippocampus and left pallidum, and between the right amygdala and left lingual gyrus. *Post hoc* Tukey test revealed significantly decreased functional connectivity in the cPTSD group for the left PCC and the right putamen, and for the right PCC and bilateral MCC when compared with both the control group and the PTSD group, respectively. Correlation analysis revealed a significant negative correlation between CAPS score and functional connectivity of the left PCC and the right pallidum, and of the right putamen and left angular gyrus, in the control group.

Statistical analysis of the psychometric scales showed that QoL scores did not differ among the control, PTSD, and cPTSD groups. Although it may be expected that control participants who did not develop PTSD or cPTSD would have better subjective QoL, we interpret this insignificant difference as reflecting the specific selection of participants. In particular, the controls were also NKDs, who were likely to have experienced traumatic events and to have low socioeconomic status, even without a clinical diagnosis of PTSD or CPTSD.^43^ The CAPS score, which reflects an individual's capacity for coping, showed significant differences between the groups. It is plausible that people who develop PTSD or cPTSD may have a lower capacity for coping than those who do not, even if they have a similar subjective feeling about their QoL. As our aim was to delineate the neural correlates that underlie PTSD and cPTSD, along with their relationships with coping capacity, it can be said that our participant groups well represented our study goal and hypothesis.

ROI-to-ROI analysis revealed functional network-level disruption of the DMN, which supports our hypothesis. More specifically, the results suggested differences in PCC-grounded functional connectivity among the three groups, with these differences involving the left angular gyrus, bilateral MCC, bilateral thalamus and basal ganglia. However, the PCC and angular gyrus constitute the DMN; significant results in the MCC, thalamus and basal ganglia require further interpretation. Unsurprisingly, a previous meta-analysis of fMRI activation in patients with PTSD indicated that abnormality of the MCC is a general finding, irrespective of the type of trauma experienced, and is involved in processing specific motor components of noxious stimuli.^44^ Another finding related to fMRI activity in PTSD was in the ROI-to-voxel analysis, which indicated a difference in functional connectivity between the right amygdala and left lingual gyrus. The lingual gyrus plays an important part in visual processing, especially visual memory,^45^ and its role in the amygdala extends to emotional visual memory processing.^46^ As expected, given the symptoms of re-experiencing PTSD, the amygdala and lingual gyrus are among the regions that show changes in resting-state activity in patients with PTSD.^47^ However, it should be noted that the ROI-to-voxel analysis results were statistically corrected with less conservative thresholds and require careful interpretation.

Whereas the above results can be thought to replicate findings for PTSD in general, aberrant functional connectivity reaching the thalamus and basal ganglia components is rather specific to our study sample, that is, NKDs. Significant results for the basal ganglia were found in both the ROI-to-ROI and ROI-to-voxel analyses, demonstrating the potential role of the basal ganglia in mediating the interplay between the subcortical-level fear circuit and functional network-level DMN. Although the basal ganglia are best known for coordinating motor functions,^48^ a more recent viewpoint is that they have a general role as coordinators of various functions, such as in the cognitive^49^ and emotional^50^ domains. Our results suggest that NKDs show unique involvement of the basal ganglia in coordinating the interaction of subcortical and functional network-level circuitry in PTSD and cPTSD.

The neural implications of PTSD and cPTSD in NKDs were further delineated using a *post hoc* Tukey test. Significant decreases in the strength of functional connectivity were found between the right PCC and bilateral MCC and between the left PCC and right putamen in the cPTSD group, compared with the PTSD and control groups. These findings suggest that prolonged and repeated exposure to traumatic events blunts the strength of the functional connectivity between the MCC and right PCC and between the left PCC and right putamen significantly more than single or no discrete trauma events. A recent study using electroencephalogram reported weaker thalamic functional connectivity in patients with child abuse cPTSD compared with PTSD and control during the inhibitory Go/NoGo task.^51^ This is in contrast to our results, where according to the ROI-to-ROI analysis, the relationship of thalamic involvement and involvement of the DMN was not specific to the cPTSD group. This discrepancy may be related to a difference in the fMRI experiment (resting state versus task) or, more interestingly, to the type of trauma (forced displacement versus child abuse). Future studies to investigate the neural effects of cPTSD based on the type of trauma could focus on the functional connectivity of the thalamus.

In the *post hoc* correlation analysis, the negative correlations of CAPS score with the functional connectivity of the left PCC–right pallidum and of the right putamen–left angular gyrus were not significant in the PTSD and cPTSD groups. These results suggest that NKDs with better coping capacity show lower connectivity strength between the DMN (left PCC and left angular gyrus) and the right basal ganglia (pallidum and putamen), whereas this relationship is blunted in NKDs who develop PTSD or cPTSD. Given that several studies have suggested that the putamen and pallidum are related to resilience and coping in response to stressful conditions,^52^^,^^53^ the interactions of the right putamen and pallidum with the DMN can be considered to have an important role in resilience and coping in NKDs, as found in the control group. However, the insignificant correlations in the PTSD and cPTSD group indicate that these disorders affect the functional connectivity between the DMN and the right basal ganglia and blunt its role in coping to stressful events. Although this interpretation might require careful observation with more relevant data, we expect that it may be clinically relevant and that functional connectivity between the DMN and the right basal ganglia could be a potential future target for neuromodulation in the treatment of PTSD and cPTSD.

One of the limitations of our study is that medication, which could have affected functional connectivity, was not considered in the analysis. Another possible limitation is that the ROI-to-voxel analysis was not statistically thresholded at the cluster level, which may have led to an overestimation of false-positive findings. Last, only female participants were included in this study to minimise the possibility of group differences in the gender ratio, which limits the generalisability of our results.

In conclusion, the results confirmed our three hypotheses regarding functional connectivity changes in traumatised NKDs. Specifically, alterations in functional connectivity related to the limbic system and the DMN were found, along with involvement of the basal ganglia. Coping and adaptation showed different correlation patterns with functional connectivity in both patients with PTSD and those with cPTSD when compared with people without PTSD. Among the strengths of our study, it is the first to investigate resting-state functional connectivity in NKDs, focusing on both PTSD and cPTSD, with a large number of prospectively recruited participants. The results provide new insight into the neurological underpinnings of PTSD and cPTSD, which is expected to help deepen our biological understanding of trauma-related disorders and improve their treatment.

## Supporting information

Kim et al. supplementary material Kim et al. supplementary material

## Data Availability

The data that support the findings of this study are available from the corresponding author, Y.-C.J., upon reasonable request. Restrictions may apply owing to privacy concerns.
